# The Impact of Writing About Gratitude on the Intention to Engage in Prosocial Behaviors During the COVID-19 Outbreak

**DOI:** 10.3389/fpsyg.2021.588691

**Published:** 2021-02-24

**Authors:** Raquel Oliveira, Aíssa Baldé, Marta Madeira, Teresa Ribeiro, Patrícia Arriaga

**Affiliations:** ^1^Department of Social and Organizational Psychology, Iscte – University Institute of Lisbon, CIS-IUL, Lisbon, Portugal; ^2^Intelligent Agents and Synthetic Characters Group (GAIPS), INESC-ID, Lisbon, Portugal

**Keywords:** COVID-19, gratitude, prosocial behavior, Portugal, Brazil, empathy, emotions

## Abstract

The ongoing coronavirus disease 2019 (COVID-19) pandemic has quickly swept the globe leaving a devastating trail of lost human lives and leading to a public health and economic crisis. With this in mind, prosociality has been heralded as a potential important factor to overcome the negative effects of the pandemic. As such, in this study, we examined the effectiveness of a brief reflexive writing exercise about recent experiences of gratitude on individuals’ intentions to engage in prosocial behaviors using a sample of 253 participants living in Portugal and 280 participants living in Brazil. Participants were randomly assigned to either a condition in which they were asked to write about recent experiences of gratitude or a control group in which they were asked to write about daily tasks. We predicted that the gratitude intervention would increase state gratitude and, consequently, increase positive affect and empathic concern, and decrease negative affect, leading to increased intentions to engage in prosocial behaviors during the COVID-19 pandemic. A moderated serial–parallel mediation analysis, in which we controlled for gender, age, and level of religiosity, indicated that our manipulation led to increases in state gratitude, which in turn increased positive emotions and empathic concern, leading to increased prosocial intentions in both countries. A content analysis of participants’ responses in the gratitude group revealed that relationships with others and health and well-being were the central themes of their gratitude experiences during the COVID-19 global pandemic.

## Introduction

In the wake of the global health crisis caused by the spread of the novel coronavirus disease 2019 (COVID-19), unprecedented public health measures have been implemented to curtail the resulting death toll and the overload of public health systems (Rajkumar, 2020). The disruption of daily routines caused by these public health measures coupled with the looming economic burden of this pandemic is all stress-inducing factors that are likely to contribute to emotional and mental distress (Pfefferbaum and North, 2020). For example, levels of depression and anxiety grew nearly two to three times higher among the general population after the COVID-19 outbreak (Ebrahimi et al., 2020), and the sensitivity to social risks increased, while levels of positive emotion and life satisfaction plummeted (Li et al., 2020). Although worrisome, these adverse effects on well-being are consistent with examinations of the psychological impacts of previous pandemics (e.g., Hawryluck et al., 2004; Lau et al., 2005) and might have consequences that outlive the pandemic (Lee et al., 2007).

Nonetheless, while disease outbreaks seem inevitable, their negative impact can be diminished (Victor and Ahmed, 2019). In this work, we seek to evaluate the effectiveness of a brief reflexive writing exercise about recent experiences of gratitude in increasing people’s intention to engage in prosocial behaviors during the pandemic. In accordance with guidelines from the World Health Organization [WHO] (2020), we acknowledge that solidarity and prosociality are essential factors in the management of the negative outcomes of the pandemic. Moreover, we argue that such exercises, although not a replacement for professional interventions, can play an essential role in regulating emotional responses, during a period in which physical distancing is encouraged, possibly hindering access to a mental healthcare system, already deficient in some countries before the pandemic (Barbato et al., 2016).

Gratitude is defined as the acknowledgment of a positive personal outcome that was not earned or deserved, but instead, freely bestowed upon the individual by others (Bono et al., 2004). Of the many ways to induce gratitude, journaling or writing about different experiences in which one felt gratitude seems to be one of the most common. This approach seems to be effective because it combines both the benefits of self-disclosure and the benefits of gratitude (for meta-analyses, see Frattaroli, 2006; Dickens, 2017, respectively). At an individual level, it can buffer stress and negative emotions resulting from traumatic events (e.g., Fredrickson et al., 2003), and is associated with increased well-being (Davis et al., 2016). At an interpersonal level, writing about gratitude has a low positive correlation with prosociality (Emmons and McCullough, 2003; Emmons and Mishra, 2011; Ma et al., 2017). In this context, prosociality has been defined as “(…) behaviors, efforts, or intentions designed to benefit, promote, or protect the well-being of another individual, group, organization, or society” (Ma et al., 2017, p.4). Theoretical approaches about the link between gratitude and prosociality suggest that gratitude can induce prosociality by (1) serving as a moral barometer (McCullough et al., 2001), (2) supporting reciprocal exchange (Nowak and Roch, 2007), and (3) favoring the construction and maintenance of interpersonal relationships (Algoe, 2012; Ma et al., 2017). Similarly, positive affect, state gratitude, and empathic concern can also be relevant predictors of prosocial behavior (Emmons and McCullough, 2003; Aknin et al., 2018; Coyne et al., 2018).

To better comprehend the role of gratitude in eliciting prosocial behavior during the pandemic, we take into consideration the potential moderating role of country of residence (Portugal and Brazil), attending to differences in the measures adopted in each country to mitigate the consequences of the pandemic^1^. In addition, considering past research demonstrating sex differences in gratitude (Kashdan et al., 2009), and the association between religiousness and prosocial behavior (Guo et al., 2020), we will also control participants’ gender and religiousness.

With this work, we seek to contribute to the efforts to contain the adverse effects caused by the COVID-19 pandemic, by leveraging the positive potential of gratitude as a platform for interpersonal support and prosociality.

## Goals and Hypotheses

The goal of this study is to evaluate the effectiveness of a brief reflexive writing exercise about gratitude on participants’ intentions to engage in prosocial behaviors during the COVID-19 pandemic.

**FIGURE 1:**
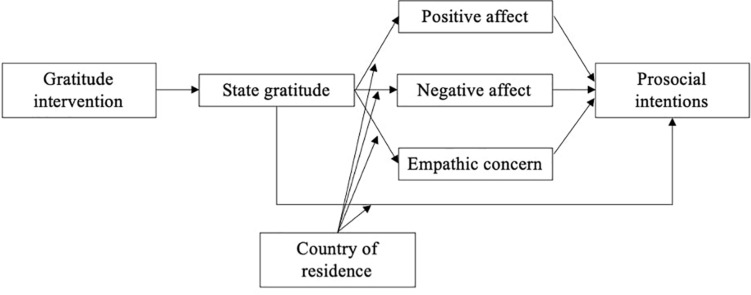
Hypothesized model linking the gratitude intervention to prosocial intentions.

In this context, we expect that writing about recent experiences of gratitude, in comparison with a control group, would contribute to increase gratitude state, which in turn will increase their intentions to behave prosocially (H1); we also expected that the effects of intervention on gratitude states and prosocial behavior would be mediated by an increase in positive affect (H2), a reduction in negative emotions (H3), and an increase in empathic concern toward vulnerable individuals to the COVID-19 (H4; see Figure 1). In addition, we explored whether the country of residence would moderate the effects of gratitude states on the outcomes (affect, empathic concern, and prosocial intentions).

In addition, we will conduct a content analysis of participants’ narratives of gratitude to explore what they felt grateful for during the pandemic period.

## Materials and Methods

### Sample

An initial convenience sample of 895 participants was collected through the dissemination of the questionnaire on online social media platforms (see Supplementary Figure 1). Of those, 362 participants were excluded because of (1) completing the survey in less than 3 min^2^ or having more than 50% of missing responses (*n* = 255), (2) not providing sociodemographic data (*n* = 42), (3) not living in Portugal or in Brazil (*n* = 31), and (4) failing to follow the instructions for the manipulation (e.g., mentioning being grateful in the control condition; *n* = 34). The final sample was composed of 533 participants (66.82% completion rate), balanced between the two conditions, of which 253 were living in Portugal and 281 in Brazil. Participant’s ages ranged between 18 and 82 years (M = 40.15, SD = 15.64); most were female (*n* = 382) and currently employed (58%). Only 18% reported being part of an at-risk professional group (i.e., professionals who kept working during the pandemic in jobs that required close contact to a large amount of people or to people potentially infected with COVID-19, e.g., nurses) and 64% reported not knowing anyone infected with COVID-19. There were no statistical differences between conditions on sociodemographic characteristics (see Supplementary Table 1).

### Manipulation

Participants were instructed to reflect for 3 min about recent experiences of gratitude (gratitude condition) or daily tasks in which they engaged (control group) during the previous week, depending on the condition they were assigned to. Then, they were instructed to write three to five sentences about those experiences, based on the instructions from Emmons and McCullough (2003) for the gratitude condition and from Bodenhausen et al. (1994) for the control condition. The full instructions given to participants in both conditions are presented in Supplementary Table 4.

### Materials and Measures

Detailed information regarding the items used in this study is presented in Supplementary Tables 4–6.

To assess affective states, we used the Portuguese version of the short form of the Positive and Negative Affective Schedule (PANAS-SF; Watson et al., 1988; Galinha et al., 2014). The PANAS-SF is composed of 10 items, five for Negative Affect and five for Positive Affect, and participants indicated to what extent they felt each emotion on a scale ranging between 1 (“Not at all”) and 5 (“Extremely”). In addition, we added three emotional states to assess state gratitude (“grateful,” “thankful,” and “appreciative”), based on Frias et al. (2011).

To investigate participants’ perception of the writing task, the following items were added: “The task made me feel better emotionally,” “I want to implement this task in my daily life,” “I will advise other people to try this task because I believe it will make them feel better emotionally,” and “The task was easy to complete.” The items were rated on a seven-point scale (1 “Strongly disagree” to 7 “Strongly agree”).

In addition, we used the three items developed by Pfattheicher et al. (2020) to assess how much empathic concern toward the most vulnerable to COVID-19 participants felt using a scale ranging between 1 (“Strongly disagree”) and 7 (“Strongly agree”; see Supplementary Table 5).

For prosocial intention, we asked participants to indicate to what extent they intended to participate in COVID-19-related prosocial behaviors during the following weeks by using two items from Li et al. (2020); e.g., “Dedicate time, donate money or supplies to chartered organizations or relevant institutes (e.g., hospitals)”; (see Supplementary Table 5) and adding three items: “Elucidate to others ways to deal with the current pandemic,” “Devote time to deliver goods and/or food to others,” and “Get in touch with others to see if they need help,” evaluated on a five-point scale (1 “Never” to 5 “Very often”).

Finally, participants were asked to indicate their sex, education level, level of religiousness, marital status, nationality, current residence, belonging to a high-risk profession, current health condition, and practice of social distancing. The items to measure the level of religiousness and current health condition were adapted from the European Social Survey (2018). Participants evaluated themselves on a 10-point scale (1 “Not at all religious” to 10 “Very religious”) on the item “Regardless of whether you belong to a particular religion, how religious would you say you are?” and on a five-point scale (1 “Very good” to 5 “Very bad”) on the item “How is your health in general?”

### Content Analysis

To analyze participants’ written experiences of gratitude, we adapted the coding scheme developed by Meier (2018). The final coding scheme included the following categories: (1) relationships with others (including spending time with others, having the presence of significant others, and receiving care, affection, and support), (2) health and well-being (including self-care, one’s own health-being and that of others, and the satisfaction of life needs), (3) work (i.e., having a good job and good colleagues), (4) personal strengths and adversity (which included mentions to one’s personal strengths and negative events), (5) leisure and time management (including enjoying nature, engaging in leisure activities, and having the liberty to manage time), (6) material possessions, and (7) pets. In addition, we included two categories: (8) God, church, and religion and (9) government, adapted from the coding scheme by Gordon et al. (2004). Given the specific context of the pandemic, we also added one category related to (10) gratitude for technological-mediated means used to keep touch with others (e.g., phone and Facebook).

To analyze participants’ responses to the gratitude exercise, two authors (MM and TR) read and coded all the responses according to the aforementioned coding scheme. Disagreements were solved through joint discussion.

### Procedure

The present study was approved by the Ethics Committee of Iscte – University Institute of Lisbon (Ref. 29/2020). Data were collected using Qualtrics between 7 and 21 April 2020. After agreeing with the informed consent, participants were asked to indicate their age and then were randomly assigned to one of the conditions (gratitude or control). Thereafter, participants responded to the gratitude scale and other positive and negative emotions, followed by the empathy and prosocial behavior scales. Then, they indicated their opinions about the task and provided sociodemographic data. Finally, participants were presented with a debriefing statement, which also included the contact of one of the authors and public health recommendations related to the COVID-19 outbreak. The survey took approximately 15 min to complete and the order of the items within each scale was randomized.

## Results

### Hypotheses Testing

All scales presented good levels of reliability (all α ≥ 0.80), and linear Pearson correlations among the main variables are presented in Supplementary Table 2.

A missing data analysis was also performed to analyze the distribution of missing values. This analysis indicated that there were no values missing at random (all Little’s MCAR > 0.05), and as such, missing values were replaced using expectation–maximization imputation. The percentage of missing items per each scale or sub-scale is presented in Supplementary Table 5.

To test our hypotheses, we conducted a moderated serial–parallel mediation analysis using the PROCESS SPSS macro (version 3.5) developed by Hayes (2018). Country of residence and gratitude state were centered before the construction of their product. In these analyses, we also controlled for age, gender, and religiosity, given their significant relation with the outcomes. Initial screening for multicollinearity among the main predictors of prosocial intentions and covariates has not shown cause for concern (variance inflation factor below the threshold of 5 and tolerance above 0.2; Hair et al., 2014). Preliminary tests of heteroscedasticity were also examined for each outcome with the Breusch–Pagan and Koenker tests using the macro Heteroskedasticity for SPSS. These tests were only statistically significant for the variance of the state gratitude errors across groups. Thus, standard errors were corrected with heteroscedasticity consistent covariance matrix. Moreover, non-parametric bootstrapping analyses were conducted (10,000 resampling) using the percentile method, with 95% CI (95CI) for all the tests in the conditional process model.

Detailed results of the moderated serial–parallel mediation model are summarized in Tables 1, 2, including statistics to all the indirect and direct effects in the analysis and the inferential test for each. Given Hayes’s (2018) recommendation to rely on bootstrap CI instead of results solely based on normal theory approach, we present both estimates.

**TABLE 1:** Moderated serial–parallel mediation of the effects of group intervention on prosocial intentions.

Antecedent	Outcomes	Coeff.	Bootstrap			*t*	SE (HC3)	*p*
	
			SE	LL	UL			
Intercept	Gratitude state (M1)	−0.75	0.12	−0.98	−0.52	−6.31	0.12	<0.001
Group (IV)		0.27	0.07	0.13	0.40	3.82	0.07	<0.001
Religiosity (C_1_)		0.06	0.01	0.04	0.09	4.74	0.01	<0.001
Gender (C2)		−0.18	0.08	−0.34	−0.02	−2.14	0.08	0.033
Age (C3)		0.01	0.002	0.002	0.01	2.83	0.002	0.005
	*R*^2^ = 0.13, *F*(4,527) = 17.34, *p* < 0.001							
Intercept	Positive affect (M2)	2.56	0.10	2.35	2.77	24.56	0.10	<0.001
Gratitude state (M1)		0.42	0.04	0.35	0.49	11.49	0.04	<0.001
Residence (W1)		−0.19	0.07	−0.32	−0.06	−2.88	0.07	0.004
Group × residence		0.04	0.07	−0.10	0.17	0.53	0.07	0.595
Religiosity (C1)		0.01	0.01	−0.01	0.03	0.77	0.01	0.441
Gender (C2)		0.23	0.06	0.11	0.35	3.79	0.06	<0.001
Age (C3)		0.01	0.002	0.004	0.01	3.80	0.002	<0.001
	*R*^2^ = 0.30, *F*(6,525) = 35.45, *p* < 0.001							
Intercept	Negative affect (M3)	2.68	0.12	2.46	2.91	23.16	0.12	<0.001
Gratitude state (M1)		−0.11	0.04	−0.19	−0.04	−3.04	0.04	0.002
Residence (W1)		0.27	0.08	0.12	0.42	3.52	0.08	<0.001
Group × residence		−0.18	0.07	−0.33	−0.04	−2.47	0.07	0.014
Religiosity (C1)		0.01	0.01	−0.02	0.04	0.78	0.01	0.437
Gender (C2)		−0.16	0.06	−0.29	−0.03	−2.45	0.07	0.015
Age (C3)		−0.02	0.002	−0.02	−0.01	−7.31	0.002	<0.001
	*R*^2^ = 0.15, *F*(6,525) = 13.42, *p* < 0.001							
Intercept	Empathic concern (M4)	6.56	0.12	6.32	6.79	54.16	0.12	<0.001
Gratitude state (M1)		0.12	0.04	0.04	0.21	2.81	0.04	0.005
Residence (W1)		0.01	0.08	−0.14	0.15	0.09	0.08	0.928
Group × residence		0.01	0.08	−0.14	0.17	0.18	0.08	0.859
Religiosity (C1)		0.02	0.02	−0.01	0.05	1.11	0.02	0.266
Gender (C2)		−0.48	0.09	−0.65	−0.31	−5.58	0.09	<0.001
Age (C3)		0.005	0.003	−0.01	0.0004	−1.76	0.003	0.078
	*R*^2^ = 0.11, *F*(6,525) = 7.68, *p* < 0.001							
Intercept	Prosocial intentions (DV)	1.10	0.38	0.37	1.86	2.88	0.38	0.004
Group		0.06	0.06	−0.07	0.19	0.94	0.07	0.348
Gratitude state (M1)		0.07	0.05	−0.03	0.16	1.38	0.05	0.168
Positive affect (M2)		0.18	0.06	0.07	0.29	3.15	0.06	0.002
Negative affect (M3)		0.02	0.05	−0.09	0.12	0.33	0.05	0.745
Empathy (M4)		0.24	0.04	0.15	0.33	5.45	0.04	<0.001
Residence (W1)		0.14	0.08	−0.01	0.29	1.86	0.08	0.064
Group × residence		−0.05	0.09	−0.23	0.12	−0.60	0.09	0.549
Religiosity (C1)		0.03	0.01	0.01	0.06	2.44	0.01	0.015
Gender (C2)		−0.34	0.08	−0.50	−0.19	−4.35	0.08	<0.001
Age (C3)		0.01	0.003	0.004	0.01	3.51	0.003	<0.001
	*R*^2^ = 0.28, *F*(10,521) = 19.51, *p* < 0.001							

*IV, independent variable (group: 0 = control; 1 = gratitude); DV, dependent variable (prosocial intentions); M1, gratitude state; M2, positive affect; M3, negative affect; M4, empathic concern; W1, moderator (residence: 0 = Portugal, 1 = Brazil); Cn, covariates; Coeff., unstandardized regression coefficient; SE (HC3), standard error (corrected with heteroskedasticity consistent covariance matrix); Boot 95 CI, 95% bootstrap confidence intervals with lower (LL) and upper (UL) bounds.*

**TABLE 2:** Moderation mediation paths according to country of residence.

Path	Residence	Effect	SE	LL	UL
Group → Gratitude state → Prosocial	Moderated mediation	−0.01	0.03	−0.07	0.03
	
	Portugal	0.03	0.02	−0.01	0.07
	Brazil	0.01	0.02	−0.02	0.05

Group → Gratitude state → Positive affect → Prosocial	Moderated mediation	0.002	0.004	−0.005	0.01
	
	Portugal	0.02	0.01	0.01	0.04
	Brazil	0.02	0.01	0.01	0.04

Group → Gratitude state → Negative affect → Prosocial	Moderated mediation	−0.001	0.003	−0.007	0.005
	
	Portugal	−0.0001	0.001	−0.002	0.002
	Brazil	−0.001	0.003	−0.01	0.01

Group → Gratitude state → Empathy → Prosocial	Moderated mediation	0.001	0.01	−0.01	0.01
	
	Portugal	0.01	0.005	0.0003	0.02
	Brazil	0.01	0.005	0.001	0.02

As shown in Table 1, the overall model, including age, gender, and religiosity as covariates, accounted for 28% of the variance in prosocial intentions, *F*(10,521) = 19.51, *p* < 0.001. Although the results indicated that there was not a significant direct effect of the intervention on prosocial intentions (*B* = 0.06, *SE* = 0.07, *t* = 0.94, *p* = 0.35), there were two significant indirect paths through state gratitude in subsequent mediator variables: one through increased overall positive affect (in both countries *B* = 0.02, 95CI Boot [0.01, 0.04]) and another through the increase in empathic concern (*B* = 0.01, 95CI Boot for Portugal [0.0003, 0.02] and 95CI Boot for Brazil [0.001, 0.02]), in both cases leading to increased prosocial intentions. In addition, we requested bootstrap estimates for a pairwise comparison between the two significant serial indirect effects supporting the effect of gratitude intervention on prosocial intentions through gratitude state on positive affect (H2) or on empathic concern (H4). The results showed no difference between the two specific indirect effects because the bootstrap CI included zero (*B* = 0.01, 95CI Boot [-0.002, 0.03]), suggesting that both mechanisms are similarly relevant in explaining the effect of gratitude states induced by the writing intervention on intentions for prosocial behaviors.

In contrast, neither the indirect path of the group intervention on prosocial intentions through state gratitude nor the path through the impact of state gratitude on negative affect was statistically significant, therefore not supporting H1 and H3. Moreover, the mediational paths were not moderated by country of residence, indicating that all the indirect results were similar for both countries.

Regarding the comparison between countries for each outcome, results indicated that participants currently living in Portugal, in comparison with those in Brazil, expressed higher positive affect but lower prosocial intentions. The effect of state gratitude on negative affect was moderated by country of residence, indicating that higher state gratitude was associated with lower negative affect for participants living in Brazil (*B* = −0.47, *SE* = 0.06, *p* < 0.001), but not for those living in Portugal (*B* = −0.02, *SE* = 0.05, *p* = 0.69).

Finally, there were also interesting results for the covariates. Women reported higher state gratitude and higher negative affect but also lower positive affect, and stronger prosocial intentions, than men. In addition, as age increased, the expression of gratitude, positive affect, and prosocial intentions tended to increase, whereas negative affect decreased. Finally, religiosity only remained positively related to state gratitude and prosocial intentions, after controlling for the other variables in the model.

### Exploratory Analyses

For the analysis of participants’ perception of the task, we grouped the responses in three categories (with disagree corresponding to the lowest three values of the scale, agree corresponding to the three highest values of the scale, and neutral corresponding to the middle point of the scale). Results revealed that most agreed that the task made them feel better (71%) and that the task was easy (87%). Moreover, most participants said they were likely to repeat the writing exercise (72%) and to recommend it to acquaintances (72%).

An independent *t*-test involving a composite variable corresponding to the joint means of the items included to measure participants’ evaluation of the task revealed that there were no differences [*t*(531) = −0.86, *p* = 0.39], suggesting that both the control (*M* = 5.44, *SD* = 1.12) and the manipulation group (*M* = 5.52, *SD* = 1.04) perceived the writing exercise very favorably.

For the content analysis, inter-coder agreement was calculated using the KALPHA macro for SPSS (Hayes and Krippendorff, 2007), and the results showed that the inter-coder reliability was excellent (Krippendorff’s α ≥ 0.91).

Detailed results (frequency and examples) of the qualitative responses given by participants in the gratitude group as well as the agreement scores and number of disagreements per category are presented in Supplementary Table 3. In total, we collected 946 gratitude statements, and each participant listed an average of 3.65 topics (*SD* = 1.26; *Min* = 1; *Max* = 8).

The gratitude sentences that were mentioned more often belonged to the categories “relationships with others” (*n* = 284) and “health and well-being” (*n* = 284). Within the first category, participants emphasized the presence of important people (*n* = 160), and within the second category, they emphasized their own well-being and that of others (*n* = 176).

Participants also reported feeling grateful for their own personal strengths and the ability to deal with negative events (*n* = 99), for their job and co-workers (*n* = 63), for their material possessions (*n* = 42), and for God (*n* = 41).

A small number of people reported feeling grateful for being able to engage in leisure activities and to manage their time more effectively during the pandemic (*n* = 35), for technology (*n* = 17), for the government’s response (which included the efforts of healthcare professionals and other essential workers; *n* = 16), and for their pets (*n* = 12).

## Discussion and Future Work

People’s ability to find things to be grateful for, even in the most adverse situations, is nothing short of remarkable. In this paper, we sought to leverage this ability by evaluating the effectiveness of a brief reflexive writing exercise in promoting prosocial behaviors during the COVID-19 pandemic.

Results suggested that our manipulation affected prosocial intentions by increasing state gratitude, which in turn led to an increase in positive affect and empathic concern, thus confirming our H2 and H4, respectively. However, state gratitude did not influence participants’ intentions to engage in prosocial behavior neither directly (H1) nor indirectly through its effect in negative affect (H3). Similarly, we did not also find a moderating effect of participants’ country of residence in these mediational paths, suggesting that the aforementioned results were identical for both participants living in Portugal and in Brazil.

Overall, our findings are congruent with past research that identifies gratitude as being an adequate target for interventions aimed at promoting prosociality and well-being (Watkins et al., 2004; Davis et al., 2016; Dickens, 2017; Ma et al., 2017). However, although adequate, gratitude interventions seem to affect only some outcomes, while leaving others unaffected. For example, our results regarding negative affect and the indirect effect of state gratitude on prosocial behavior are congruent with the findings of a recent meta-analysis, in which the authors found mixed results for the effects of gratitude in negative affect and no substantive effects in prosocial behavior. Much of this is likely related to the type of comparison activity employed, as previous studies have demonstrated that gratitude interventions are more effective when compared with negative exercises (such as writing about daily hassles), but less effective when compared with neutral (e.g., listing daily tasks) or other positive activities (e.g., writing about things that make one happy; Davis et al., 2016; Dickens, 2017).

In addition, although our manipulation led to increased positive emotions resulting in higher prosocial intentions, there may have been benefits stemming from a consistent application of grateful thinking or journaling that our manipulation did not allow us to capture. The work of O’Connell et al. (2017) supports this assertion by demonstrating that both the consistency and the rate of gratitude journaling might modulate the positive effects of gratitude on well-being. In this sense, we would like to call for future work investigating the potential positive outcomes of writing gratitude interventions applied consistently during long stretches of time. We hypothesize that such interventions, coupled with other psychological exercises, might serve to mitigate, to some extent, the present and long-term negative effects of the COVID-19 pandemic and to promote prosocial and adaptive responses.

Moreover, previous studies hint at the hypothesis that the effects of gratitude interventions might be influenced by other variables that were not taken into account in this study. For example, although interventions like gratitude journaling that involve the recall of past experiences are very common in literature, some studies show that they induce weaker effects when compared with *in vivo* manipulations (Ma et al., 2017). Similarly, studies that investigate generalized gratitude (as in this study) also tend to display lower associations with prosocial behavior in comparison with studies that investigate gratitude targeted at specific people or deeds (Ma et al., 2017). As such, future studies or applications of gratitude writing exercises should consider these aspects, and emphasize reciprocal, specific, and when possible, *in vivo*, inductions of gratitude.

Furthermore, although in this study we decided to investigate the effects of gratitude in prosocial behavior among the general population, we would like to call for more studies directed at investigating the impacts of gratitude journaling in specific groups of people who are more susceptible to the negative psychological impacts of the pandemic and look at different possible positive outcomes within those. For example, due to the strain put on healthcare workers during the pandemic and the resulting stress, this professional group has been identified as being at an increased risk for mental health problems (Greenberg et al., 2020), and hence, the proactive implementation of psychological strategies that diminish this risk is a necessary and important next step (Duan and Zhu, 2020). In addition, recent research has also emphasized the negative consequences of the pandemic (and associated restrictions) to the mental health of the population in general (e.g., stress), and future research is necessary to tackle the issue of the possible role of gratitude in improving or protecting individual’s mental health from the negative effects of this pandemic (Duan and Zhu, 2020).

## Conclusion

Prosociality is a topic of interest to all, especially in the midst of a global pandemic. Despite the recent development of vaccines, at this time, the bulk of the effort to limit the spread of COVID-19 and of its negative consequences is still in the hands of all of us. This can include following the WHO health protective guidelines, such as wearing a protection mask, which has been found to be related to prosocial behaviors (Campos-Mercade et al., 2020) or checking up on others who are more vulnerable to COVID-19, to loneliness or to mental illness. Our results suggest that engaging in writing exercises about recent experiences of gratitude can increase state gratitude, which in turn increases other positive emotions and empathic concern, providing a do-it-yourself, cost-effective strategy to increase prosocial behaviors during the pandemic.

## Data Availability Statement

The raw data supporting the conclusions of this article will be made available by the authors, without undue reservation.

## Ethics Statement

The studies involving human participants were reviewed and approved by Ethics Committee of Iscte – University Institute of Lisbon (Ref. 29/2020). The participants provided their written informed consent to participate in this study.

## Author Contributions

All authors contributed to the conceptualization, investigation, development of the methodology, data collection, formal analysis, validation, visualization, writing, reviewing, and editing of the original and final draft. PA also supervised the work and curated the data.

## Conflict of Interest

The authors declare that the research was conducted in the absence of any commercial or financial relationships that could be construed as a potential conflict of interest.

## Footnotes

1 In Portugal, an emergency state was declared on March 18, with confinement and physical social distancing being mandatory (Diário da República, 2020). In Brazil, the government advised social distancing, but a state of emergency was not declared and confinement was not imposed (Ministério da Saúde, 2020). Differences in the strictness of government responses to the pandemic (e.g., school closures, workplace closures, and travel bans) between countries were also reported. According to the Government Response Stringency index (Hale et al., 2020), Portugal showed a higher stringency index (82.41) compared with Brazil (77.31) between January and April 2020.

2 Three minutes was used as an exclusion criterion as this was the amount of time that participants were asked to reflect for during the writing exercise (see Supplementary Table 4 for the full instructions).
