# Supplementary material for: The Impact of Writing About Gratitude on the Intention to Engage in Prosocial Behaviors During the COVID-19 Outbreak
Source: Front Psychol. 2021 Feb 24;12:588691. doi: 10.3389/fpsyg.2021.588691 (PMC7943462; doi:10.3389/fpsyg.2021.588691)
Supplement: Supplementary file 1 [file Table_1.pdf]

frontiers

# *Supplementary Material*

## Table of Contents

*Figures*

Figure 1. Flowchart depicting the selection of participants for the study. 2

*Tables*

Table 1. Group comparison of sociodemographic variables. 3

Table 2. Pearson linear correlations among the main variables and reliability. 5

Table 3. Coding scheme used for participants' recent experiences of gratitude and inter-coder agreements. 6

Table 4. Instructions given to participants regarding the writing exercise (Portuguese and English translation). 11

Table 5. Survey items (Portuguese and English translation). 13

Table 6. Sociodemographic questions (Portuguese and English translation). 17

Figure 1. Flowchart depicting the selection of participants for the study.

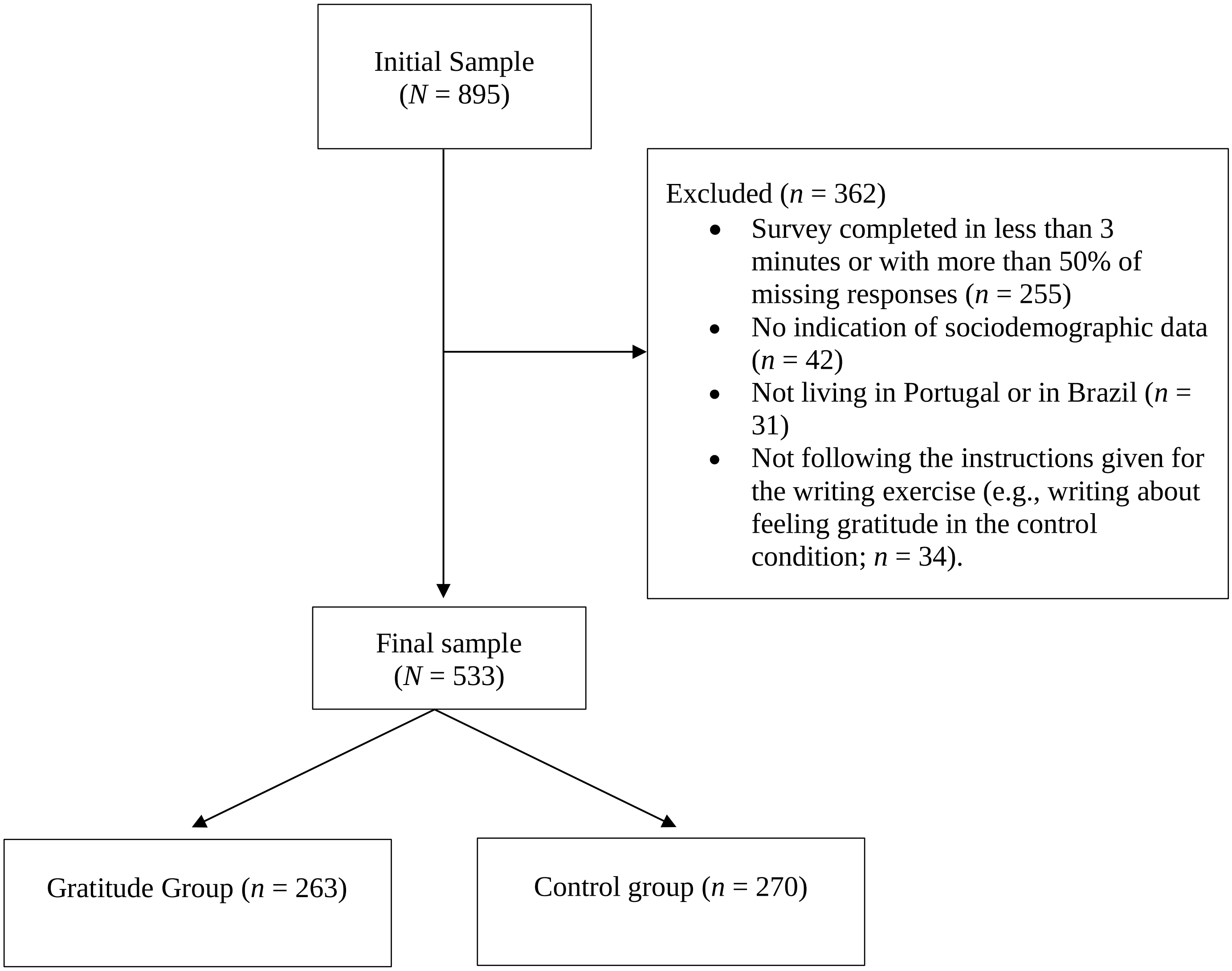

Table 1. Group comparison of sociodemographic variables.

| Variables | Group | | | | | | | |
|---|---|---|---|---|---|---|---|---|
| | Total | | Gratitude | | Control | | $\chi^2$ | *p* |
| | *N* | % | *N* | % | *N* | % | | |
| Gender | | | | | | | 0.99 | .32 |
| Women | 382 | 72 | 194 | 74 | 188 | 70 | | |
| Men | 150 | 28 | 69 | 26 | 81 | 30 | | |
| Total | 532 | 100 | 263 | 100 | 269 | 100 | | |
| Residence | | | | | | | 0.83 | .36 |
| Portugal | 253 | 48 | 117 | 45 | 136 | 50 | | |
| Brazil | 280 | 52 | 146 | 55 | 134 | 50 | | |
| Total | 533 | 100 | 263 | 100 | 270 | 100 | | |
| Nationality | | | | | | | 1.83 | .16 |
| Portugal | 243 | 46 | 111 | 43 | 132 | 49 | | |
| Brazil | 285 | 54 | 147 | 57 | 138 | 51 | | |
| Total | 528 | 100 | 258 | 100 | 270 | 100 | | |
| Confinement | | | | | | | 0.15 | .90 |
| Yes | 459 | 86 | 226 | 86 | 233 | 86 | | |
| No | 74 | 14 | 37 | 14 | 37 | 14 | | |
| Total | 533 | 100 | 263 | 100 | 270 | 100 | | |
| Days in confinement | | | | | | | 0.88 | .35 |
| ≥ 30 days | 390 | 87 | 197 | 89 | 193 | 86 | | |
| ≤ 31 days | 57 | 13 | 25 | 11 | 32 | 14 | | |
| Total | 447 | 100 | 222 | 100 | 225 | 100 | | |
| Belonging to an at-risk group for getting COVID-19 | | | | | | | 1.85 | .17 |

| Variables | Group | | | | | | | |
|---|---|---|---|---|---|---|---|---|
| | Total | | Gratitude | | Control | | $\chi^2$ | *p* |
| | *N* | % | *N* | % | *N* | % | | |
| Yes | 97 | 18 | 45 | 17 | 52 | 20 | | |
| No | 433 | 82 | 223 | 83 | 210 | 80 | | |
| Total | 530 | 100 | 268 | 100 | 262 | 100 | | |
| Has acquaintances with COVID-19 | | | | | | | 0.38 | .86 |
| Yes | 192 | 36 | 96 | 36 | 96 | 36 | | |
| No | 340 | 64 | 172 | 64 | 168 | 64 | | |
| Total | 532 | 100 | 268 | 100 | 264 | 100 | | |
| | *M* | *SD* | *M* | *SD* | *M* | *SD* | *t* | *p* |
| Age | 40.15 | 15.64 | 40.01 | 15.41 | 40.30 | 15.90 | -0.21 | .84 |
| General health | 1.84 | 0.71 | 1.81 | 0.71 | 1.88 | 0.70 | 1.11 | .27 |

*Note*. General health was measured on a 5-point scale (1 “Very good” - 5 “Very bad”), with higher values indicating lower levels of health.

Table 2. Pearson linear correlations among the main variables and reliability.

| | 1 | 2 | 3 | 4 | 5 | 6 | 7 | 8 | 9 | 10 |
|---|---|---|---|---|---|---|---|---|---|---|
| 1 Group | -- | | | | | | | | | |
| 2 Gender | -.04 | -- | | | | | | | | |
| 3 Residence | .06 | -.03 | -- | | | | | | | |
| 4 Age | .01 | -.02 | .40*** | -- | | | | | | |
| 5 Religiosity | -.01 | -.15** | .45*** | .39*** | -- | | | | | |
| 6 State gratitude | .16*** | -.14** | .10* | .21*** | .28*** | **.81** | | | | |
| 7 Positive Affect | .10* | .07 | -.01 | .23*** | .16*** | .50*** | **.81** | | | |
| 8 Negative Affect | -.02 | -.09* | .06 | -.28*** | -.03 | -.16*** | -.26*** | **.78** | | |
| 9 Empathic Concern | -.03 | -.29*** | .02 | -.03 | .11* | .17*** | .04 | .17*** | **.80** | |
| 10 Prosocial Intentions | .07 | -.27*** | .22*** | .28*** | .39*** | .29*** | .24*** | -.02 | .31*** | **.81** |
| *M* | | | | 40.15 | 6.24 | 3.60 | 2.99 | 2.08 | 6.35 | 3.71 |
| *SD* | | | | 15.64 | 2.96 | 0.86 | 0.74 | 0.72 | 0.80 | 0.86 |

*Note*. $*p < .05$, $**p < .01$, $***p < .001$. *p*s are two-tailed; Reliability coefficients (Cronbach's alpha) are in bold and displayed along the diagonal. Group coded 0 = Control; 1 = Gratitude; Gender coded 0 = Women, 1 = Men; Residence coded 0 = Portugal, 1 = Brazil. These values were calculated using estimated means (EM). Religiosity and empathic concern were measured on a 7-point scale, with higher values indicating higher levels of religiosity and empathic concern, respectively. Positive affect, negative affect, state gratitude and prosocial intentions were measured using a 5-point scale, with higher estimated means corresponding to higher levels of positive affect, negative affect, state gratitude and prosocial intentions, respectively.

Table 3. Coding scheme used for participants' recent experiences of gratitude and inter-coder agreements.

| Category | Sub-category | Definition | Examples | Kripendorff's alpha (KALPHA) | Number of disagreements |
|---|---|---|---|---|---|
| Relationships with others (*n* = 284) | Spending time with others (*n* = 63) | Being grateful for the opportunity to spend more time with one or more specific person(s), in person or through video calls. | "*Gratitude for being able to spend more time with my son.*" | .98 | 1 |
| | Presence of important people (*n* = 160) | Being thankful for the presence of a specific person in ones' life, including being grateful for the love, companionship, and friendship one receives from that important person. | "*Thankfulness for the fantastic relationship with my husband because in these days of isolation, although we are grumpy, we are still great friends and looking out for each other's needs.*" | .98 | 2 |
| | Receiving support from others (*n* = 40) | Being thankful for the support that one receives from others during the confinement and social distancing period. This can include psychological support, financial support, and helpful gestures. | "*Gratitude for being able to count on psychological support in this moment.*" | .96 | 2 |
| | Receiving care or affection from others (*n* = 21) | Being grateful for receiving affection or appreciation from others. This can include receiving a message | "*I felt grateful for the phone calls and messages from friends that showed concern in knowing how I am doing.*" | 1.00 | -- |

| Category | Sub-category | Definition | Examples | Kripendorff's alpha (KALPHA) | Number of disagreements |
|---|---|---|---|---|---|
| | | or call or other gestures that express love and concern. | | | |
| Health and well-being (*n* = 284) | Self-care (*n* = 31) | Felling grateful for having more time to take care of ones' health and well-being during the confinement. | "*I feel grateful for being able to rest more.*" | .98 | 1 |
| | Own health and well-being (*n* = 88) | Gratefulness for ones' physical and mental health and well-being during the pandemic period. This also includes the possibility to be confined safely and comfortably at home. | "*I feel grateful for being healthy and safe.*" | .96 | 2 |
| | Health and well-being of others (*n* = 88) | Being grateful for the health and well-being of significant others. | "*I feel grateful for not having any loved ones infected.*" | 1.00 | -- |
| | Life Needs (*n* = 77) | Feeling thankful for being alive, for having a good financial income to pass through this vulnerable time and for life needs being fulfilled e.g. enough food and water; love; etc. | "*I feel gratitude for having economic conditions that allow me to manage the social isolation at home without concerns of that matter.*" | .96 | 2 |
| Work (*n* = 63) | Good co-workers (*n* = 8) | Being thankful for having supportive, tight and helpful colleagues or boss during the pandemic. | "*I feel grateful for having co-workers that recognize and praise my work, even at a distance.*" | .91 | 1 |

| Category | Sub-category | Definition | Examples | Kripendorff's alpha (KALPHA) | Number of disagreements |
|---|---|---|---|---|---|
| | A good job (*n* = 55) | Thankfulness for the opportunity to work from home, for having a nice job, and for being able to maintain ones' job during the pandemic. | "*I feel grateful that I can keep my job during this moment of crisis and that I am able to do it from my home.*" | .99 | 1 |
| Personal strengths and adversity (*n* = 99) | Personal strengths (*n* = 32) | Thankfulness for the opportunity to know your personal strengths better in such a difficult and challenging time. This includes the ability to use psychological resources, being resilient, autonomous, personal growth, etc. | "*I feel grateful for my positivity.*" | .93 | 4 |
| | Negative Events (*n* = 67) | Being thankful for the positive things that you can extract from this negative period. This also includes person´s capability to help others among this period. | "*I feel grateful for the moments of self-reflection during the lockdown and understanding what is really important in my life.*" | .93 | 4 |
| Leisure and time management (*n* = 35) | Enjoying nature and other abstract things (*n* = 16) | Being grateful for nature and abstract things, such as the birds chirping, the sun, art, music, etc. | "*I feel grateful for being able to hear the birds singing from my window*." | 1.00 | -- |
| | Free time activities (*n* = 17) | Being grateful for the extra time one has to engage in leisure activities (e.g., sports, reading a book, cooking, ride a bike, etc). | "*I feel grateful because I can spend more time practicing physical exercise (football).*" | .94 | 3 |

| Category | Sub-category | Definition | Examples | Kripendorff's alpha (KALPHA) | Number of disagreements |
|---|---|---|---|---|---|
| | Time Management (*n* = 17) | Appreciation for having more time flexibility to manage one's schedule. | "*I feel gratitude for being able to manage my time in a more efficient way, mostly on a professional level, because even though we are connected there's a feeling of being able to set priorities more easily*." | .97 | 1 |
| Material possessions (*n* = 42) | Objects of material value (*n* = 42) | Being thankful for having objects of material value that are self-evident for most people. | "*I feel grateful for having a house.*" | .94 | 2 |
| Pets (*n* = 12) | Pets (*n* = 12) | Gratefulness for the opportunity to spend more time with pets, or for being able to enjoy their company and affection. | "*I feel grateful for having a puppy that keeps me distracted during these days*." | 1.00 | -- |
| Religion (*n* = 41) | God, Church, Religion (*n* = 41) | Being grateful for the presence of God in one's life. This also includes being grateful for one's religion and church for helping one cope with this difficult time. | "*Gratitude to God for the opportunity I am receiving during the lockdown, to reflect on my spiritual difficulties.*" | 1.00 | -- |
| Institutional response (*n* = 16) | Government's response (*n* = 2) | Being grateful for the effectiveness of the government's' response to the pandemic outbreak. | "*To the government for their job in solving the problems.*" | 1.00 | -- |
| | Health Care Providers (*n* = 14) | This also includes gratitude for health care providers who help those infected with COVID-19, and other essential workers who | "*I feel grateful towards all the professionals who try to stop the pandemic every day.*" | .92 | 2 |

| Category | Sub-category | Definition | Examples | Kripendorff's alpha (KALPHA) | Number of disagreements |
|---|---|---|---|---|---|
| | | provide services during the lockdown. | | | |
| Technology (*n* = 17) | Technological devices (*n* = 17) | Felling of gratitude for technological devices (e.g., phone, internet) and for other technological-mediated means that can be used to keep touch with others (e.g., Facebook, WhatsApp). | "*I feel grateful for the internet that allows me to see my friends and family who are far away from me.*" | .97 | 1 |
| Others (*n* = 53) | Others (*n* = 53) | Being thankful for things that are not directly related to the pandemic context (e.g. your education) or thankful for other people's values (e.g. see that people are respecting the norms imposed, etc.) | "*I feel grateful for people coming together as a community to work towards a purpose.*" | .91 | 7 |

Table 4. Instructions given to participants regarding the writing exercise (Portuguese and English translation).

| Instructions | Group | Portuguese | English |
|---|---|---|---|
| Manipulation | Gratitude group | Há situações e pessoas nas nossas vidas pelas quais podemos sentir-nos grato(a)s. A gratidão é caracterizada pelo reconhecimento e apreciação do que há de positivo no mundo e nas pessoas. Pode também ocorrer nos momentos em que achamos ter recebido algo positivo de forma inesperada e altruísta. Podemos sentir-nos gratos em relação a pessoas, fontes espirituais e religiosas, e até animais. São exemplos de gratidão as seguintes frases: “Sinto-me grato(a) por poder passar mais tempo com a família”, “Sinto-me grato(a) por ter mais tempo para mim”, "Sinto-me grato porque a pessoa X ajudou-me". Pedimos-lhe que reflita durante 3 minutos acerca das experiências pelas quais se sente grato(a) durante esta fase de pandemia, ao longo da última semana. Na tarefa seguinte, pedimos-lhe que partilhe essas suas reflexões pessoais de gratidão. Se terminar antes desse tempo, pode avançar para a tarefa de escrita. Por favor, escreva 3 a 5 frases sobre experiências de gratidão que pensou na tarefa anterior. | There are moments and people in our lives we feel grateful for. Gratitude can be characterized as a feeling of recognition and appreciation for the positive side of people and the world. It can also occur in moments where we think we received something positive in an unpredictable way. We can feel grateful for people, spiritual and religious sources, and even animals. Examples include: “I feel grateful for being able to spend time with my family”, “I feel grateful for having more time to myself”, “I feel grateful because X helped me”.<br>Please reflect for 3 minutes about experiences you felt grateful for during the current pandemic, that happened last week. In the following task, we ask you to share your personal gratitude reflections.<br>Please write 3 to 5 sentences about those gratitude experiences. |
| | Control group | Nesta fase de pandemia há várias tarefas e atividades rotineiras em que nos envolvemos. São exemplos de tarefas e atividades rotineiras as seguintes frases: “fiz o pequeno almoço quase todos os dias", "lavei roupa", "arrumei a casa com regularidade", "preparei refeições", "limpei a casa".Pedimos-lhe que reflita durante 3 minutos acerca de tarefas e/ou atividades rotineiras que tenha realizado durante esta fase de pandemia, ao | During the current pandemic, there are several daily tasks we get involved in. Examples of these tasks are “I made breakfast everyday”, “I washed clothes”, “I cleaned the house regularly”, “I prepared meals”.<br>Please reflect for 3 minutes about tasks or daily activities you performed during the past week. In the following task, we ask you to share those activities or tasks. |

| Instructions | Group | Portuguese | English |
|---|---|---|---|
| | | longo da última semana. Na tarefa seguinte, pedimos-lhe que partilhe essas tarefas ou atividades rotineiras. Se terminar antes desse tempo, pode avançar para a tarefa de escrita.<br>Por favor, escreva 3 a 5 frases sobre as tarefas ou atividades que pensou na tarefa anterior. | Please write 3 to 5 sentences about those tasks or activities. |

Table 5. Survey items (Portuguese and English translation).

| Construct | Portuguese | English | Items | % of missing responses | Scale |
|---|---|---|---|---|---|
| Positive Affect | Por favor, indique a intensidade com que sente cada uma das emoções neste momento. | Please indicate the intensity with which you feel each of the following emotions right now. | 1. Determined (*Determinado/a)*<br>2. Active (*Ativo/a)*<br>3. Proud (*Orgulhoso/a)*<br>4. Inspired (*Inspirado/a)*<br>5. Interested (*Interessado/a)* | 0.6-0.9% | 1. Not at all (*Nada)*<br>2. A little (*Um pouco)*<br>3. Moderately (*Moderadamente)*<br>4. Very much (*Bastante)*<br>5. Extremely (*Extremamente)* |
| Negative affect | Por favor, indique a intensidade com que sente cada uma das emoções neste momento. | Please indicate the intensity with which you feel each of the following emotions right now. | 1. Nervous (*Nervoso/a)*<br>2. Scared (*Assustado/a)*<br>3. Tormented (*Atomentado/a)*<br>4. Irritable (*Irritado/a)*<br>5. Disgusted (*Repulsa*) | 0.7-1.1% | 1. Not at all (*Nada)*<br>2. A little (*Um pouco)*<br>3. Moderately (*Moderadamente)*<br>4. Very much (*Bastante)*<br>5. Extremely (*Extremamente)* |
| State gratitude | Por favor, indique a intensidade com que sente cada uma das emoções neste momento. | Please indicate the intensity with which you feel each of the following emotions right now. | 1. Grateful (*Grato/a)*<br>2. Thankful (*Agradecido/a)*<br>3. Appreciative (*Apreço)* | 0.9-1.1% | 1. Not at all (*Nada)*<br>2. A little (*Um pouco)*<br>3. Moderately (*Moderadamente)*<br>4. Very much (*Bastante)*<br>5. Extremely (*Extremamente)* |
| Prosocial Intentions | Indique em que medida pretende realizar cada um seguintes comportamentos de | Indicate to what extent you plan to engage in the following helping | 1. Donate time, donate money or supplies to chartered organizations or relevant institutions (e.g., hospitals). | 0-0.6% | 1. Never (*Nunca/Não pretendo)*<br>2. Seldom (*Raramente)*<br>3. Sometimes (*Algumas vezes)* |

| Construct | Portuguese | English | Items | % of missing responses | Scale |
|---|---|---|---|---|---|
| | ajuda nos próximos tempos. | behaviors in the near future. | (*Dedicar tempo, fazer donativos ou dar bens materiais para apoiar organizações ou instituções relevantes (ex. hospitais)).*<br>2. Help others understand how to deal with the situation and prevent the spread of COVID-19. (*Ajudar as pessoas a perceber como podem lidar com a situação e prevenir a propagação do COVID-19).*<br>3. Elucidate to others ways to deal with the current pandemic. (*Esclarecer as pessoas sobre formas de lidar com a situação atual da pandemia).*<br>4. Devote time to deliver goods and/or food to people. (*Dedicar tempo a entregar bens e/ou comida a pessoas).*<br>5. Get in touch with other people to see if they need help. (*Entrar em contato com outras pessoas para verificar se precisam de ajuda).* | | 4. Often (*Frequentemente)*<br>5. Very often (*Muitas Vezes)* |

| Construct | Portuguese | English | Items | % of missing responses | Scale |
|---|---|---|---|---|---|
| Empathic concern | Por favor, para cada frase indique o seu grau de concordância ou discordância. | Please indicate how much you agree or disagree with the following sentences. | 1. I am very concerned about those most vulnerable to COVID-19. (*Estou preocupado com os mais vulneráveis à COVID-19).*<br><br>2. I feel compassion for those most vulnerable to COVID-19. (*Sinto compaixão pelos mais vulneráveis à COVID-19).*<br><br>3. I am quite moved by what can happen to those most vulnerable to COVID-19. (*Sinto-me emocionado por aquilo que pode acontecer aos mais vulneráveis à COVID-19).* | 0 | 1. Strongly disagree (*Discordo fortemente)*<br>2. Disagree (*Discordo)*<br>3. Somewhat disagree (*Discordo ligeiramente)*<br>4. Don't disagree nor agree (*Não discordo nem concordo)*<br>5. Somewhat agree (*Concordo ligeiramente)*<br>6. Agree (*Concordo)*<br>7. Strongly Agree (*Concordo fortemente)* |
| Participant's perception of the task | Com base na tarefa de escrita que realizou, indique o seu grau de concordância ou discordância com cada frase. | Regarding the writing task, please indicate how much you agree or disagree with each sentence. | 1. The task made me feel better emotionally. (*A tarefa fez-me sentir melhor emocionalmente).* | 1.1-1.3% | 1. Strongly disagree (*Discordo fortemente)*<br>2. Disagree (*Discordo)*<br>3. Somewhat disagree (*Discordo ligeiramente)* |

| Construct | Portuguese | English | Items | % of missing responses | Scale |
|---|---|---|---|---|---|
| | | | 2. I will implement this task in my daily life because it helps me to feel better emotionally. (*Pretendo vir a enquadrar esta tarefa no meu dia-a-dia porque me ajuda a sentir melhor emocionalmente).*<br><br>3. I will advise other people to try this task because I believe it will make them feel better emotionally. (*Pretendo vir a enquadrar esta tarefa no meu dia-a-dia porque me ajuda a sentir melhor emocionalmente).*<br><br>4. The task was easy to complete. (*Foi fácil realizar a tarefa).* | | 4. Don't disagree nor agree (*Não discordo nem concordo)*<br>5. Somewhat agree (*Concordo ligeiramente)*<br>6. Agree (*Concordo)*<br>7. Strongly Agree (*Concordo fortemente)* |

Table 6. Sociodemographic questions (Portuguese and English translation).

| Topic | Portuguese | English | Scale |
|---|---|---|---|
| Belonging to an at-risk group | Faz parte de algum grupo profissional em maior risco (e.g. profissional de saúde, farmacêutico, técnico de diagnóstico e terapêutica, força de segurança, trabalho um supermercado, lar, entre outros)? | Are you part of any at-risk professional group (e.g. healthcare professional, pharmacist, diagnostic and therapeutic technician, security force, supermarket employee, among others)? | 1. Yes (*Sim)*<br>2. No (*Não)* |
| General health | Como avalia a sua saúde em geral? | How is your health in general? | 1. Very Good (*Muito boa)*<br>2. Good (*Boa)*<br>3. Moderate (*Razoável)*<br>4. Bad (*Má)*<br>5. Very bad (*Muito má)*<br>6. I don't know (*Não sei)* |
| Acquaintances with COVID-19 | Quantas pessoas suas conhecidas foram infetadas com a COVID-19? (escreva um número aproximado, contando consigo; se não conhece, coloque "0"). | How many people do you know that have been infected by the COVID-19? (Write an approximate number counting with you; If you don´t now, put "0"). | Open-ended answer |
| Practice of social distancing | Encontra-se de momento em situação de confinamento /distanciamento físico ou social a partir da sua casa? | Are you currently in physical or social confinement/distancing from your home? | 1. Yes. For how many days? (*Sim. Por quantos dias?)*<br>2. No (*Não)* |
| Level of religiousness | Independentemente de pertencer a uma religião em particular,numa escala de 1 a 10, diria que é uma pessoa... | Regardless of whether you belong to a particular religion, how religious would you consider you are? | 1. Not religious at all (*Nada religioso/a)*<br>2. 2<br>3. 3<br>4. 4<br>5. 5<br>6. 6<br>7. 7<br>8. 8 |

| | | | |
|---|---|---|---|
| | | | 9. 9<br>10. Very religious (*Muito religioso/a)* |
| Sex | *Qual é o seu sexo?* | What is your sex? | 1. Feminine (*Feminino)*<br>2. Masculine (*Masculino)*<br>3. Other (*Outro)*<br>4. Rather not answer (*Prefiro não responder)* |
| Education level | Qual é o grau de escolaridade mais elevado que já cumpriu? | What is the highest level of education you have completed? | 1. Less than primary school (*Inferior ao 1º ciclo do ensino básico)*<br>2. Primary school (*1º ciclo do ensino básico)*<br>*3.* Second cycle of Basic Education (*2º ciclo do ensino básico)*<br>*4.* Third cycle of Basic Education (*3º ciclo do ensino básico)*<br>5. High School (*Ensino Secundário)*<br>6. Higher Education (*Ensino Superior)* |
| Nationality | Qual é a sua nacionalidade? | What is your nationality? | 1. Portuguese (*Portuguesa)*<br>2. Brazilian (*Brasileira)*<br>3. Other (*Outra)* |
| Country of residence | Em que país reside na atualidade? | In which country do you currently live? | 1. Portugal (*Portugal)*<br>2. Brazil (*Brasil)*<br>3. Other (*Outra)* |
| Marital status | *Qual é o seu estado civil?* | What is your marital status? | 1. Married (or non-marital partnership) (*Casado/a (ou em união de facto)*<br>2. Widowed (*Viúvo/a)*<br>3. Divorced (*Divorciado/a)*<br>4. Separated (married but living apart) (*Separado/a, casado mas separado/ não vive com o cônjuge)*<br>5. Single/ Unmarried (*Solteiro/ Nunca casou)* |
